# A Collection of Remarkable Cases in Surgery

**Published:** 1857-11

**Authors:** 


*A Collection of Remarkable Cases in Surgery.* By PAUL F. EVE, M. D., Professor of Surgery in the Medical Department of the University of Nashville.

Any one who should suppose that he has had remarkable cases in surgery, should glance at some of the cases cited in the work now before us, amputation of a supernumerary head, swallowing open jack-knives, pairs of suspenders, silk handkerchiefs, etc., etc. All the journals, abstracts, reviews, text-books and note-books have been diligently ransacked by Prof. Eve, for remarkable cases, until he has now a volume of eight hundred and fifty pages filled with marvel upon marvel. In the preface the author distinctly defines what he has done, claiming no more credit than is justly his due, namely, the credit of collecting in a substantial form, the records of such remarkable cases as would, perhaps, pass away, and at all events would be available to very few members of the profession. In his own words, "The volume now offered to the profession contains little else than a collection of remarkable cases in surgery. This is about all it professes to be. And believing that uncommon events and strange circumstances can best be described by those who have observed them, the language of the writer has generally been adopted, with full credit to the source whence derived. The collector has been studious to do injustice to no one. He has taken the liberty occasionally to abbreviate articles; in some instances to give them a mork striking and appropriate title; and now and then to add a few brief comments." Again: "In a work where we have found so much said for us, the labor has been light; and the only merit, if there be any at all, is in the selection of matter; of this the profession must judge."

The author has arranged the cases under appropriate heads, so that cases of injury or disease affecting any particular part of the body, may be easily found. He devotes chapters to the head, spinal column, face, neck, chest, abdomen, pelvis, genito-urinary organs, extremities, and one to miscellaneous cases. By this arrangement one is enabled to find readily any particular case, if it be in the book. This will be especially useful to teachers of surgery who should wish to quote any illustrative case which they might not be able to find elsewhere. We may congratulate ourselves that the labor of collecting and arranging these cases has been performed by one whose experience and judgment render him so competent to the task.

This work is published by J. B. Lippincott & Co., of Philadelphia, and for sale by Phinney & Co. The typography is excellent, but the few wood cuts, though they may serve to illustrate, certainly do not beautify the book.

